# Assessment of ZTD Derived from COSMIC Occultation Data with ECWMF, Radiosondes, and GNSS

**DOI:** 10.3390/s22145209

**Published:** 2022-07-12

**Authors:** Naifeng Fu, Mingbo Jiang, Fenghui Li, Peng Guo, Chunping Hou, Mengjie Wu, Jianming Wu, Zhipeng Wang, Liang Kan

**Affiliations:** 1 School of Marine Science and Technology, Tianjin University, Tianjin 300072, China; nffu@tju.edu.cn; 2 Beijing Institute of Applied Meteorology, Beijing 100029, China; qxilcs@163.com; 3 School of Electrical and Information Engineering, Tianjin University, Tianjin 300072, China; hcp@tju.edu.cn (C.H.); zpwang@tju.edu.cn (Z.W.); kllarajiayou@tju.edu.cn (L.K.); 4 Shanghai Astronomical Observatory, Chinese Academy of Sciences, Shanghai 200030, China; gp@shao.ac.cn (P.G.); mjwu@shao.ac.cn (M.W.); wujm@shao.ac.cn (J.W.); 5 University of Chinese Academy of Sciences, Beijing 100049, China

**Keywords:** zenith tropospheric delays (ZTDs), COSMIC, ECWMF, GPS-ZTD

## Abstract

Global Navigation Satellite System (GNSS) signals generate slant tropospheric delays when they pass through the atmosphere, which is recognized as the main source of error in many spatial geodetic applications. The zenith tropospheric delay (ZTD) derived from radio occultation data is of great significance to atmospheric research and meteorology and needs to be assessed in the use of precision positioning. Based on the atmPrf, sonPrf, and echPrf data from the Constellation Observing System for Meteorology, Ionosphere, and Climate (COSMIC) Data Analysis and Archiving Center (CDAAC) from 1 January to 31 December 2008 and 2012, we obtained the ZTDs of the radio occultation data (occZTD) and the corresponding radiosonde (sonZTD) and ECWMF data (echZTD). The ZTDs derived from ground-based global positioning system (GPS) observations from the International GNSS Service (IGS) were corrected to the lowest tangent point height of the matched radio occultation profile by the barometric height formula (gnsZTD). The statistical results show that the absolute values of the bias between occZTD and echZTD, sonZTD, or gnsZTD are less than 5 mm, and the standard deviations are approximately 20 mm or less, indicating that occZTD had significant accuracy in the GNSS positioning model even when the local spherical symmetry assumption error was introduced when the Abel inversion algorithm was used to obtain the refractive index profile of atmPrf. The effects of the horizontal/vertical matching resolution and the variation in the station height/latitude on the biases of occZTD and gnsZTD were analyzed. The results can be used to quantify the performance of radio occultation data for tropospheric delay error correction in dynamic high-precision positioning.

## 1. Introduction

Today, radio signals are widely used in space geodetic techniques, such as the Global Navigation Satellite System (GNSS), Doppler Orbitography Radiopositioning Integrated by Satellite (DORIS), Very Long Baseline Interferometry (VLBI), and Interferometric Synthetic Aperture Radar (InSAR) [1,2,3]. When the radio signals travel through the atmosphere, both the phase and the doppler of radio waves at the receiver change significantly. The path delay induced by the ionosphere may reach 10 m in the zenith direction and may exceed 50 m at a 5° elevation angle. Dual-frequency observations can be combined to eliminate ionospheric effects. The tropospheric delay of the global positioning system (GPS) signal near the ground is approximately 2–20 m above a 10° elevation angle, which cannot be corrected by dual-frequency observation because the neutral atmosphere is a non-dispersive medium for the frequencies of GNSS signals. Therefore, the zenith tropospheric delay (ZTD) is recognized as the main source of error in many spatial geodetic applications, and has a great impact on navigation, positioning, and its applications [4,5,6,7,8,9].

Many researchers have used the ZTD derived from meteorological data to verify the ground-based ZTD (GNSS-ZTD) measured by GNSS and have concluded that they are basically consistent with each other [4,10]. From the comparison of the ZTD calculated from the European Center for Medium Range Weather Forecast (ECMWF) by the integration method at GPS stations with that from GPS stations of the China Crustal Movement Observation Network (CMONOC), Chen found that the bias ranged from 11.5 to −28.6 mm with a corresponding average of −10.5 mm, while the largest root mean square of difference (RMSD) was 35.4 mm with an average of 24.3 mm [4], which supports the feasibility and reliability of computing the tropospheric delays and establishing the ZTD prediction model over China for navigation and positioning with ECMWF and National Centers for Environmental Prediction (NCEP) data. Other researchers have also used high-precision GNSS-ZTD to verify the ZTD derived from data obtained by many meteorological observation systems [11,12]. When evaluating the accuracy of the precipitable water (PW) from ground-based GPS, Liou found that GPS-sensed PW agreed with the dual-channel water vapor radiometer (WVR) observations with root mean square error (RMSE) accuracy of 1–2 mm, and the best agreement in PW between Microwave Radiometer and Radiosondes was approximately 2.2 mm for the nine baseline cases [13]. At the same time, GPS-ZTD can be used to construct an empirical model of ZTD related to time and location for precision point positioning (PPP). The Shanghai Astronomical observatory tropospheric delay model—Extended (SHAtropE) was developed based on the ZTD time series of the continuous GNSS sites from CMONOC and GNSS sites of surrounding areas [9]. SHAtropE was applied in the static PPP. The convergence time of GPS-only and BDS-only solutions was reduced by 8.1% and 14.5%, respectively, compared to the hybrid neutral atmosphere delay model-3 of University of New Brunswick (UNB3m) model, and the reductions were 6.9% and 11.2%, respectively, for the Global Pressure and Temperature-3(GPT3) model [14,15].

Radio occultation (RO) data are suitable for tropospheric observations due to their global coverage, high vertical resolution, and generally uniform distribution from a climatological perspective [16]. The RO observations from Constellation Observing System for Meteorology, Ionosphere, and Climate (COSMIC)-1 and those from the recently launched COSMIC-2 and other constellations have been applied to numerical weather forecast models [17]. The introduction of open-loop occultation signal tracking technology in COSMIC-1/-2 has improved the low-level atmospheric detection capability of radio occultation technology [18,19]. This makes it possible to obtain more than 40% of events with a bottom detection height less than 1 km, and the atmospheric refractive index with higher accuracy in the lower atmosphere. Many scholars use the atmospheric occultation profile with moisture information from RO products to evaluate its total precipitable water based on multiple data sources: Ground-Based GNSS, Radiosonde, Microwave Satellite, and Numerical Weather Prediction (NWP) reanalysis data [20,21]. When the atmosphere grid model is used as the background field, one-dimensional variational assimilation introduces the error from the limitation of the time and space resolution of that grid model into wetPrf which is the one-dimensional variational assimilation result between the refractive index from the occultation observation and the atmosphere background field.

Now, more and more satellite constellations in low orbit of earth with the improved GNSS RO instruments are planned or carried out, such as COSMIC-2, GeoOpt, Korea Multi-Purpose Satellite-5(KOMPSAT-5), Metop-A/-B/-C, PAZ, SPIRE, TerraSAR-X (TSX), TanDEM-X (TDX), and so on, which would greatly improve the time and space resolution of GNSS-RO events. However, the atmospheric three-dimensional information provided by GNSS RO should be used in more ways, such as improving precision positioning. Although the Numerical Weather Model (NWM) analysis field data can be used as tropospheric data sources, there are deviations at the bottom, and the horizonal resolution of grid is only up to 25 km. The existing IGS stations or Continuously Operating Reference Stations (CORS) can only provide corrections in the continental area, and they are also unable to properly correct the position of aircraft and airships at a special height. However, the ZTD around the world at a specific height can be derived by integrating the retrieved refractivity index of GNSS-RO from the specific height to the top of the troposphere. The ZTD from GNSS-RO can continue to increase its distribution resolution as low-orbit satellites continue to increase to improve precision positioning independently or as supplements of the tropospheric data sources such as NWM data and GNSS-ZTDs. For facilitating the use of RO data to enhance precision positioning, it is necessary and meaningful to verify ZTDs from the dry atmosphere profiles of GNSS-RO with that from GNSS-ZTD, as well as analyze the influence on ZTDs’ accuracy of the matching constraints such as the temporal, horizonal, and vertical distance.

Based on ZTDs from ECWMF (echZTD), radiosondes (sonZTD), and GNSS (gnsZTD), we carried out the assessment of ZTDs obtained by the occultation technique (occZTD). First, we present the four ZTD data sources and calculation methods, especially the ZTD correction method based on the barometric height formula for the height of the GNSS-ZTD which does not match the bottom tangent point height of the RO data. Then, we present the differences of ZTD (δZTD) between echZTD, sonZTD, gnsZTD, and occZTD, and present the spatial characteristics of the difference between the matched data of occZTD and echZTD (${\delta \mathrm{ZTD}}_{1}$) and the difference between the matched data of occZTD and gnsZTD (${\delta \mathrm{ZTD}}_{3}$) due to the station distribution and the matching constraints. Finally, conclusions are given.

## 2. Materials and Methods

The number of occultation events of COSMIC-1 reached the maximum in June 2007 and continued until May 2010; after rising in 2011, it rose again in 2012 and then fell to the end of its life. The years 2008 and 2012 can be used as ideal time periods for the accuracy verification of the occZTD data of COSMIC-1 in the early and late stages. The COSMIC-2 satellite brought about a sharp rise in occultation events, which reached more than 5000 in May 2020. However, CDDAC/COSMIC does not provide radiosonde data matched with COSMIC-2’s occultation profiles. A huge workload is required to obtain the precision between the occZTD and sonZTD of COSMIC-2, as one year of radiosonde needs to be collected and matched. The occZTD in the middle and low latitudes may be affected by more humidity, which may produce larger errors compared with gnsZTD, and cannot be used as a reference for the comparison results of COSMIC-1. Using the data from 2008 and 2012 (the GNSS-RO data have been reprocessed by introducing the new inversion algorithm) is the current best solution.

### 2.1. ZTDs from RO, ECMWF, and Radiosondes

The RO method obtains the refractive index of the earth’s atmosphere with a horizontal resolution of about 300 km and a vertical resolution of ~1 km by measuring the phase delay caused by the GNSS radio signal recorded in low earth orbit [22]. The COSMIC is one of the most important scientific occultation projects in recent years, providing more than 1500 atmospheric and ionospheric RO observations every day in its early days [23,24], making significant contributions to operational numerical weather forecasting and ionospheric research [25]. The COSMIC Data Analysis and Archiving Center (CDAAC) is a comprehensive data center responsible for data processing for COSMIC and several other occultation missions. The site provides users RO data at different processing levels. In this work, the atmospheric parameter profiles retrieved from atmospheric occultation data and their matched files co-located with the ECMWF high-resolution reanalysis (ECH) model and radiosonde observations were obtained from CDAAC/UCAR for 1 January to 31 December in the years of 2008 and 2012 [26]. In CDAAC, these atmosphere profiles are marked as “atmPrf”, “echPrf”, and “sonPrf”, respectively. The atmPrf files from CDAAC/COSMIC did not provide humidity information. The echPrf files from CDAAC/COSMIC provided water vapor pressure. The sonPrf file from CDAAC/COSMIC contains temperature, pressure, and moisture profiles generated from the NCAR mass store, dataset 353.4. Radiosondes do not continuously observe high-altitude atmospheric properties. The location of an occultation event may not coincide with the location of a high-altitude observation station. Therefore, temporal and spatial matching is required. In the process of data matching, the temporal resolution is 1 h, and the spatial resolution is 300 km.

Specifically, in the calculations of occZTD, sonZTD, and echZTD, the effective range of height and sampling interval of the obtained data are not consistent. Since the influence of ZTD is closely related to height, (1) we select the common height part of the profile and use the cubic spline interpolation method to interpolate the refractive indexes to the uniform height region and resolution (δh = 1 m); and (2) we integrate the refractive indexes to obtain ZTDs via Equation (1):

(1) $$\mathrm{ZTD}=10^{-6}\times \sum^{\;}\mathrm{Ref}\left(h\right)\delta h,\;$$

to reduce the error caused by the height inconsistency.

### 2.2. ZTDs from Ground-Based GNSS-ZTD

Ground-based GNSS-ZTD observation data are from the International GNSS Service (IGS) products [27], and named ZPD files, which contain the total zenith tropospheric delay, the north-south and east-west gradient components of the delay, and the root mean square error. The meteorological parameters (such as the air pressure (hPa), temperature (K), and relative humidity (%)) are obtained from the IGS stations in Rinex format. It is difficult to continuously measure from radiosonde due to its high cost and disposable equipment, but observations from ground based GNSS can provide continuous observations throughout the year.

The Saastamoinen model is read as [4,28]:

(2) $$\mathrm{ZTD}=0.002277\frac{P_{0}+\left(0.05+\frac{1255}{T_{0}+273.15}\right)e}{f\left(\varphi ,H\right)},\;$$

(3) f(φ,H)=1−0.0026cos2φ−0.00028H, 

where ZTD, e are the zenith troposphere delay (m) and wet pressure (mbar), respectively; $P_{0},T_{0},\varphi ,H$ are the dry atmospheric pressure (hPa), temperature (K), and geographic latitude (degree), altitude (km) at the GPS receiver, respectively. f (φ, H) is the vertical total mass center of the atmosphere, and only related to latitude and altitude.

The ZTD derived from ground-based GNSS data of IGS is corrected to the lowest tangent point height of the matched occultation data by the press-height formula, and the corrected ZTD data with the bottom height $H_{\mathrm{occ}}$ of occultation event can be obtained, which are named gnsZTD. Since the meteorological observation data of GNSS stations can only provide the surface temperature and pressure and relative humidity, but not the profile with temperature, humidity, and pressure along the height, it is difficult to obtain accurate ZTD changes from $H_{\mathrm{occ}}$ to the ground. For 40% of the detection depth of COSMIC-1 is less 1 km, we assume that the temperature gradient of this part is fixed:

(4) $$T\left(h\right)=T_{0}-L\left(h-H\right),$$

in which L is temperature laps rate, $6.5\left(K\cdot {\mathrm{km}}^{-1}\right),$ and $T_{0}$, H are referenced to the above explanations. In turn, the temperature at a special height h can be expressed with the temperature at the GPS receiver from ground-based observation or ECWMF model. Then, based on the pressure formula read as:

(5) $$\frac{\partial P}{\partial h}=\frac{\mathrm{Pg}}{R_{d}T\left(h\right)},\;$$

the pressure $P_{\mathrm{occ}}$ at $H_{\mathrm{occ}}$ can be obtained by integration from ground to $H_{\mathrm{occ}}$. In (5), g is the gravity coefficient;${\;R}_{d}$ is $287\left(J\cdot {\mathrm{kg}}^{-1}\cdot K^{-1}\right)$. Finally, the pressure at $H_{\mathrm{occ}}$ is used to obtain occZTD via (2) with the assumption that the ZTD at $H_{\mathrm{occ}}$ has the same proportional coefficient to $P_{\mathrm{occ}}$ as the ground ZTD to its pressure. Equation (5) with the height correction of hydrostatic delay is applied, since the atmPrf file only provides dry temperature and dry pressure. Bottom-down corrections introduce errors into the corrected ZTD if the humidity is provided by the ECMWF, and there is an increased reliance on ECMWF for atmPrf in applications when the height is below the atmPrf’s bottom. We will try to correct the height deviation of the occultation base with other data in the future.

Since the bottom heights of atmPrf, echPrf, and sonPrf are all the bottom height of the atmosphere profile from the occultation data, we corrected the ground-based ZTD data to this height based on the above correction formula. Therefore, we unified the ZTD obtained in different ways into the same height range for comparison.

However, not every IGS station provides meteorological observations, not to mention complete meteorological observations throughout the day. Moreover, there is no guarantee that the temporal resolution of the meteorological observation data at each station is the same. For this reason, when performing data correction to obtain gnsZTD, we used temperature, humidity, and pressure at GPS receiver from ECH model as the meteorological observation that this station should provide. Meanwhile, the locations of the occultation events do not usually coincide with the locations of the IGS observation stations; we needed to perform a spatial matching (the horizontal resolution of 500 km and the vertical resolution of 5 km) to generate matched dataset between GNSS-ZTD and RO ZTD. The matched dataset was used in the following studies with a filter of a special horizontal or vertical resolution.

## 3. Results and Discussions

**Figure 1:**
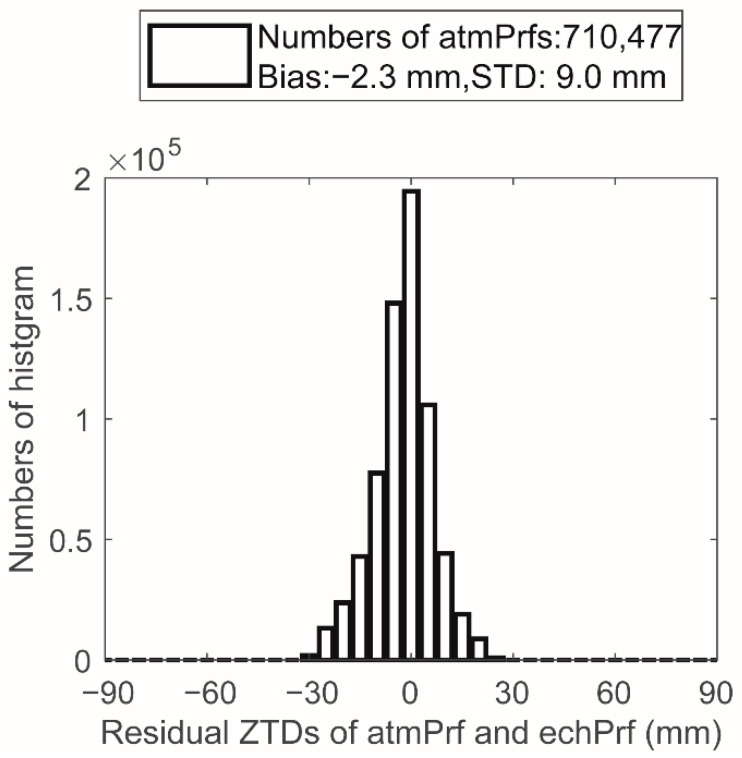
ZTD differences between matched atmPrf and echPrf in 2008.

Between 1 January and 31 December 2008, more than 710,500 effective RO events occurred, with an average of about 2000 RO events per day having accurate inversion results. The atmospheric physical parameters corresponding to each event in this experiment were obtained by interpolation of the ECH model, so the number of events counted during this time period is consistent with the number of successfully retrieved atmospheric profiles. Due to the high credibility of the ECH model results, we use the 3 sigmas principle to remove events with occZTD deviating greatly from the corresponding echZTD value. The problematic data are less than 1%. There were 710,477 data points in the final comparison between occZTD and echZTD. Figure 1 shows the statistical diagram of the difference δ${\mathrm{ZTD}}_{1}$ between the matched occZTD and echZTD data. It can be seen from Figure 1 that δ${\mathrm{ZTD}}_{1}$ is normally distributed with a bias of −2.3 mm, indicating that the refractive index of the occultation data is smaller than that of the ECMWF model; the standard deviation is 9.0 mm.

**Figure 2:**
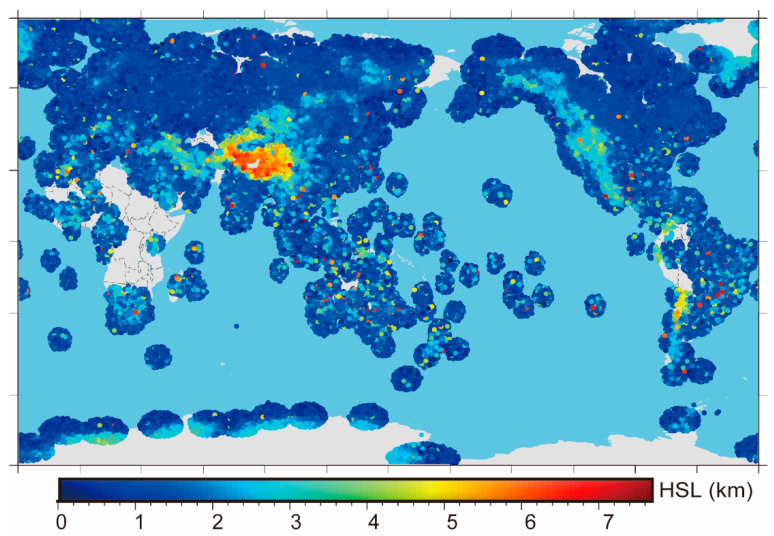
Bottom height distribution of sonPrf matched with atmPrf in 2008. The color indicates the lowest point of the common height of matched events in HSL.

**Figure 3:**
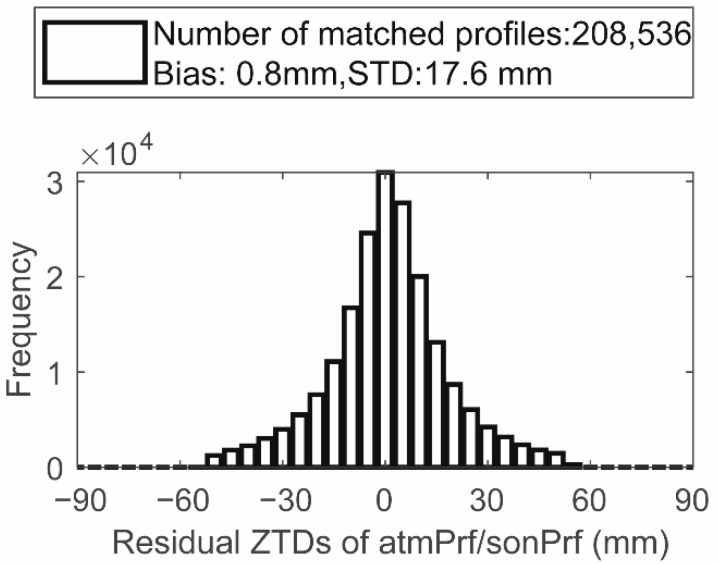
ZTD differences between matched atmPrf and sonPrf in 2008.

Figure 2 shows the global distribution of the matching results of occZTD and sonZTD, where the color indicates the lowest point of the common height of matched events in HSL, which is consistent with the distribution of the surface elevation. Figure 3 shows the statistical diagram of the difference ${\delta \mathrm{ZTD}}_{2}$ between the matched occZTD and sonZTD data. Similar to the data preprocessing of occZTD and echZTD, we removed the unreliable occZTD and sonZTD data pairs by the 3 sigmas principle. The final number of statistical points is 208,536, which is one-third of the ${\delta \mathrm{ZTD}}_{1}^{\prime }s$ number. ${\delta \mathrm{ZTD}}_{2}$ is normally distributed (the bias is 0.8 mm; the standard deviation is 17.6 mm, which is greatly higher than that of ${\delta \mathrm{ZTD}}_{1}$). This phenomenon is mainly due to the temporal and spatial inconsistency between the occZTD and sonZTD data.

**Figure 4:**
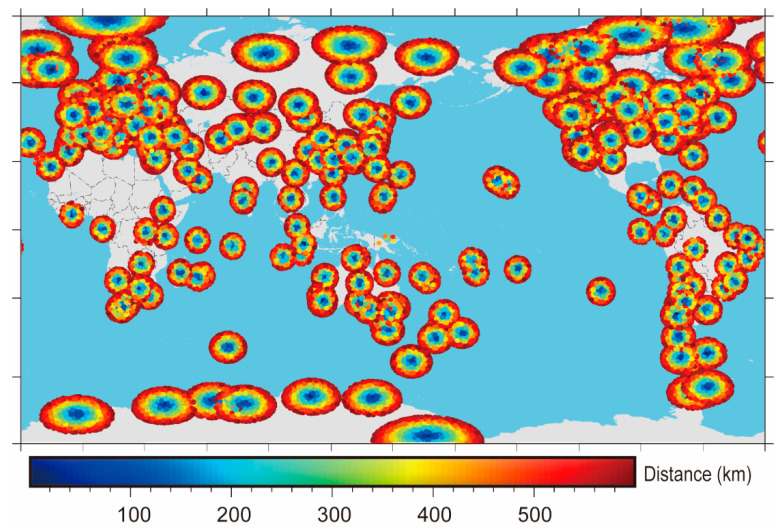
Horizontal distance distribution of gnsZTD matched with atmPrf in 2008.

**Figure 5:**
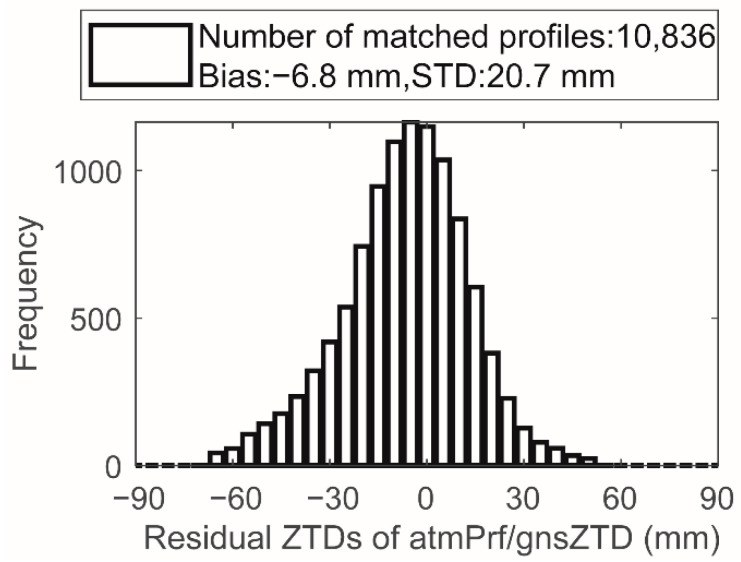
Statistics and distribution of residual ZTDs for matched atmPrf and gnsZTD in 2008.

The global distribution map of generated matched dataset introduced in Section 2 is shown in Figure 4, where the color indicates the horizontal distance (δr,km) between the lowest point of each occultation profile and the GPS observation station. To verify the consistency of ZTDs from occultation data and the ground based GNSS, a filter of a horizontal distance of 300 km and a vertical distance of 1 km was reused for the statistics of the differences between GNSS-ZTD and RO ZTD. Figure 5 shows the statistical diagram of the difference ${\delta \mathrm{ZTD}}_{3}$ between the matched data of occZTD and gnsZTD. Similar to the data preprocessing of occZTD and sonZTD, we removed the unreliable occZTD and gnsZTD data pairs by the 3 sigma principle. ${\delta \mathrm{ZTD}}_{3}$ includes 24,278 statistical points normally distributed (bias is −4.6 mm; standard deviation is 20.5 mm), but the bias and standard deviation are higher than those of ${\delta \mathrm{ZTD}}_{1}$ or ${\delta \mathrm{ZTD}}_{2}$. Although the ZTD of the solution from the ground based GNSS station has been greatly improved after correction, the ZTD is still very sensitive to the vertical distance (δh,km) between the lowest point of each occultation profile and the GPS observation station, which is discussed in the following subsection.

From the comparison of the ZTDs of 2008 and 2012 in Table 1, we can find that the accuracy of the data in these two years is similar. The standard deviation (STD) of ${\delta \mathrm{ZTD}}_{1}$,${\;\delta \mathrm{ZTD}}_{2}$, and ${\delta \mathrm{ZTD}}_{3}$ are within 10 mm, 20 mm, and 30 mm, respectively. Compared with 2008, the number of matches in 2012 is smaller, and its STD is slightly higher. Chen showed that the results of comparing GPS-ZTD and ECMWF-ZTD calculated by the integration method over China are −10.5 ± 24.3 mm [4]. By estimation with ${\delta \mathrm{ZTD}}_{1}$ and ${\delta \mathrm{ZTD}}_{3}$ in 2008, there is about 2.3 ± 22.3 mm between gnsZTD and echZTD at the bottom of the occultation profile.

From above, we can see that δZTD differs with each other, the absolute value of the bias is within 12 mm, and the standard deviation is approximately 25 mm or less. Based on the above validation, we discuss the spatial characteristics of ${\delta \mathrm{ZTD}}_{1}$ and ${\delta \mathrm{ZTD}}_{3}$ due to the station distribution and the matching process.

### 3.1. Residual Variations in Matched ZTDs between occZTD and echZTD

**Figure 6:**
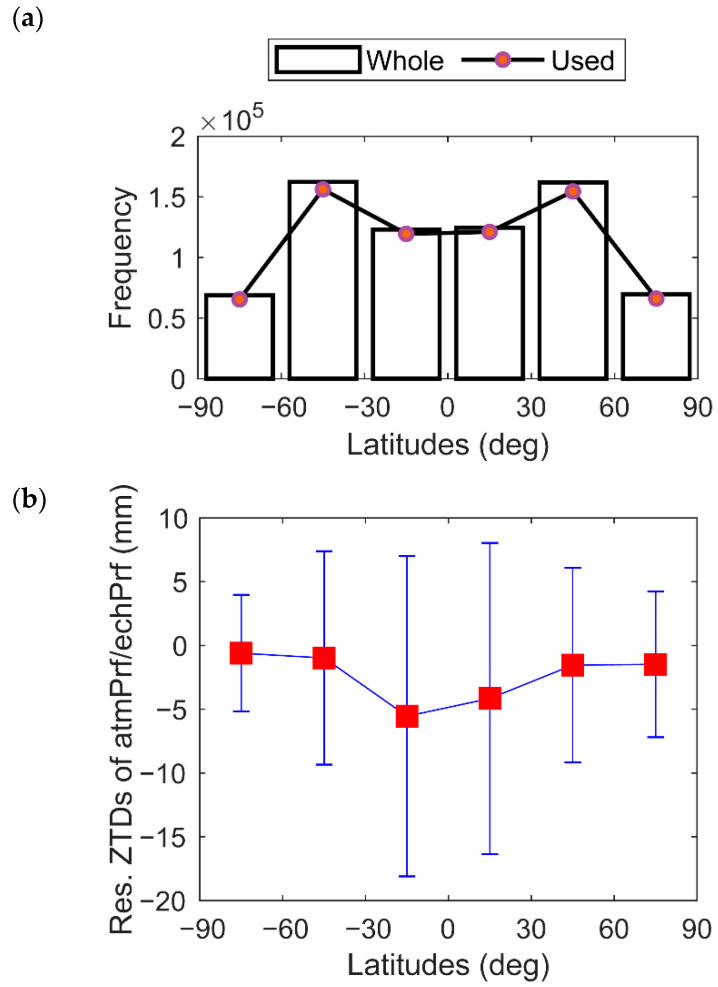
Latitudinal variations in 2008 of (**a**) the number of occultation events; and (**b**) residual ZTDs of matched atmPrf and echPrf.

The ECMWF model has better accuracy in the Northern Hemisphere than in the Southern Hemisphere [29,30]. The ZTD is affected by temperature, which has a strong relationship with latitude. To this end, we calculated the accuracy of ${\delta \mathrm{ZTD}}_{1}$ in different latitude regions. Figure 6a shows the total amount of data in each region and the amount of data involved in the statistics after deducing the gross error (3 sigmas principle). The occultation data are evenly distributed in the Northern and Southern Hemispheres and are mainly concentrated in the middle and low latitudes (approximately 80%). Figure 6b shows the trend of ${\delta \mathrm{ZTD}}_{1}$ with latitude. In the northern and southern high-latitude areas (latitudes greater than 60°), due to the low temperature and low water vapor content, the bias and standard deviation of ${\delta \mathrm{ZTD}}_{1}$ are extremely small, and their absolute values are all less than 5 mm. As the latitude decreases, the temperature and water vapor content increase, causing the ZTD error to increase. In particular, the standard deviation near the equator reaches more than 12 mm. In addition, the bias of the ZTD in the northern equatorial region is slightly smaller than that in the southern equatorial region, which verifies that the Northern Hemisphere ECMWF model can match the occultation observation data better than the Southern Hemisphere model.

**Figure 7:**
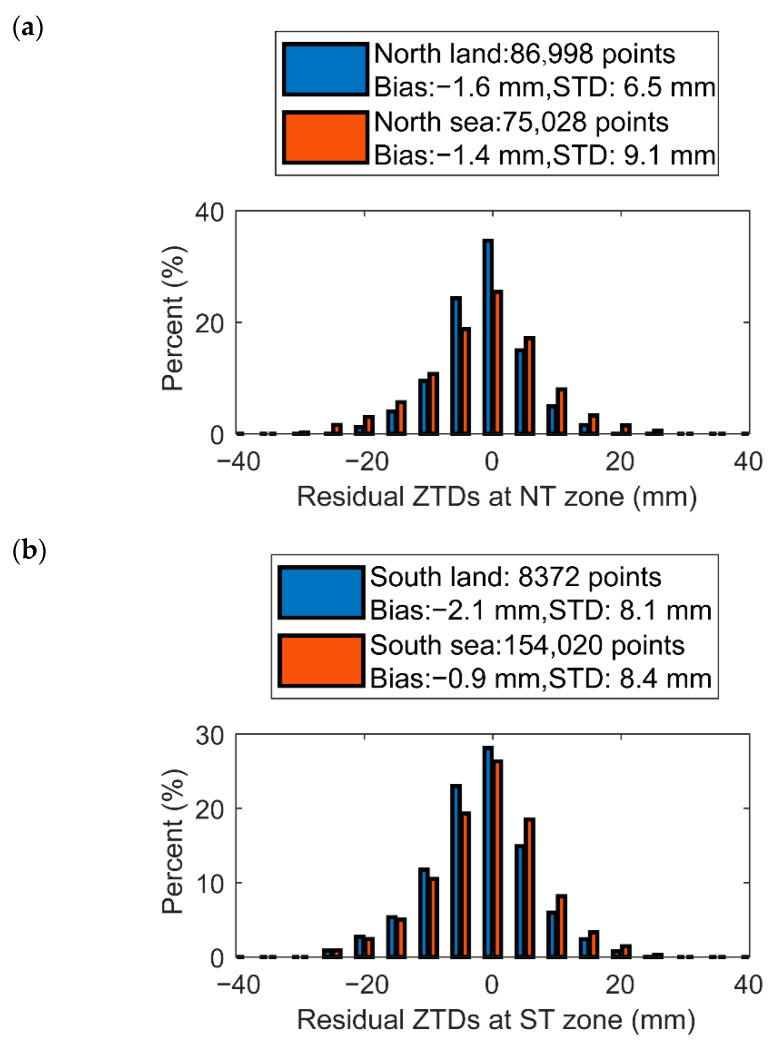
Statistics and distribution of residual ZTDs of atmPrf and echPrf in 2008 in (**a**) the south temperate (ST) zone; and (**b**) the north temperate (NT) zone. Blue indicates land and red indicates sea.

To verify whether this phenomenon is related to the distribution of land and sea, Figure 7 showed the different accuracies of ${\delta \mathrm{ZTD}}_{1}$ in the land and sea regions of the Northern and Southern Hemispheres. Figure 7a shows that in the Northern Hemisphere, the ${\delta \mathrm{ZTD}}_{1}$ difference on the land has a normal distribution with an average value of −1.6 mm and a standard deviation of 6.5 mm, which is more consistent than that in the sea. Figure 7b shows that the Southern Hemisphere land precision with standard deviation of 8.1 mm is slightly better than the Southern Hemisphere marine precision with standard deviation of 8.4 mm, but the land precision is still worse in the Southern Hemisphere than in the Northern Hemisphere. Compared with land, the ocean area has fewer atmospheric instruments to obtain surface reflectivity, temperature, humidity, and pressure, etc., so that the accuracy of the ECH model in the ocean area is not as high as that of the land area, which reduces the precision of ${\delta \mathrm{ZTD}}_{1}$.

### 3.2. Residual Variations in Matched ZTDs between occZTD and gnsZTD

When comparing occZTD and gnsZTD, two kinds of errors need to be considered. One is the error caused by the matching process. Since gnsZTD can provide a continuous time-stable ZTD, the matching error mainly comes from the uncertainty of the spatial position. This paper analyzes the spatial variation characteristics of ${\delta \mathrm{ZTD}}_{3}$ caused by horizontal and vertical matching threshold values. The other is the ZTD errors affected by the locations of GPS stations. This section analyzes the characteristics of ${\delta \mathrm{ZTD}}_{3}$ variations related to the altitude and latitude distribution of GPS stations.

**Figure 8:**
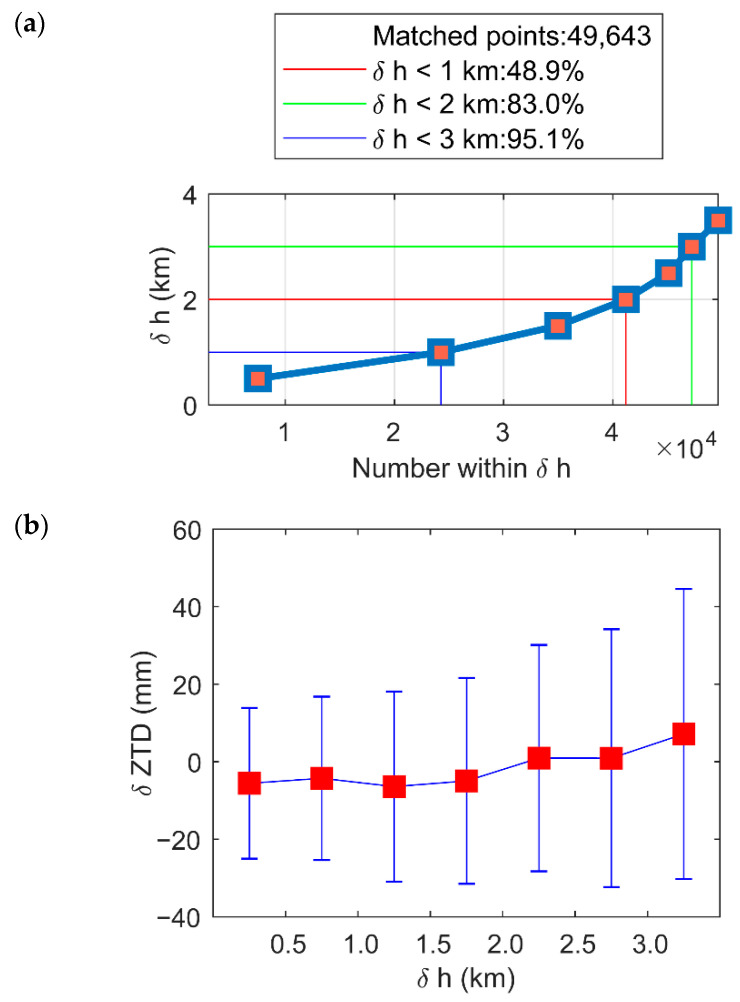
δh’s variations in (**a**) frequency and (**b**) residual ZTDs of matched atmPrf and gnsZTD in 2008. Red square mark biases of residual ZTDs of matched atmPrf and gnsZTD in δh ranges, and blue error bars in *y*-axis indicate STDs. Red circles mark the location of the *x*-axis ticks.

To analyze the effect of the error caused by the inconsistency in the vertical height between the lowest point of the occultation profile and the ground station, ${\delta \mathrm{ZTD}}_{3}$ obtained by a filter with a horizontal distance of 300 km was analyzed. Figure 8a shows the number of occultation observations and the height difference (δh,km) between the lowest level of matched RO profiles and GNSS stations. It can be seen from Figure 8a that because COSMIC uses open-loop data tracking, the percent of ${\delta \mathrm{ZTD}}_{3}$ with the δh within 1 km reaches 48.9%, and that with the δh within 3 km reaches more than 90%. Figure 8b shows the statistical precision of different δhs. Because ZTD is sensitive to δh, when δh is in the range of (0.0,3.0) km, its standard deviation increases.

**Figure 9:**
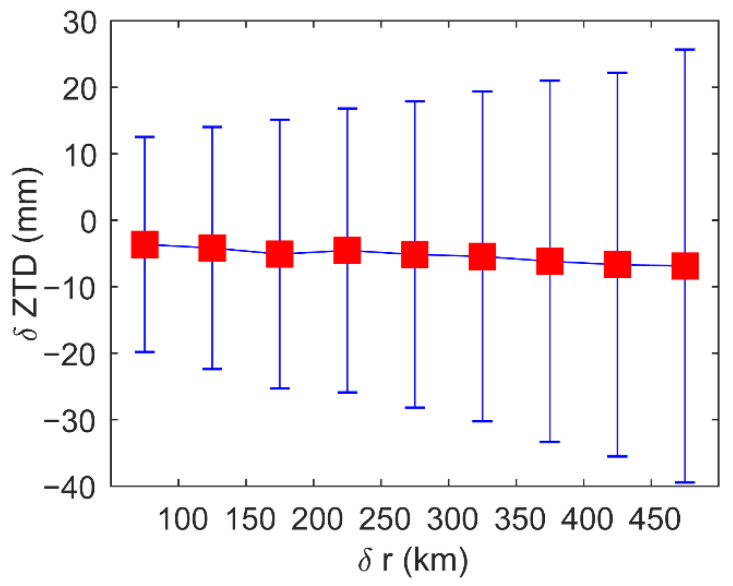
δr’s variations in residual ZTDs of matched atmPrf and gnsZTD in 2008. Red square mark biases of residual ZTDs of matched atmPrf and gnsZTD in δr ranges, and blue error bars in *y*-axis indicate STDs. Red circles marked the location of the *x*-axis ticks.

We used ${\delta \mathrm{ZTD}}_{3}$ obtained by a filter with a vertical distance of 3 km to analyze the error caused by the horizontal distance (δr,km) between the lowest point of the occultation profile and the GPS station. Figure 9 shows that as δr increases, the bias does not change significantly, but the standard deviation increases from 16.2 mm to 33.8 mm.

**Figure 10:**
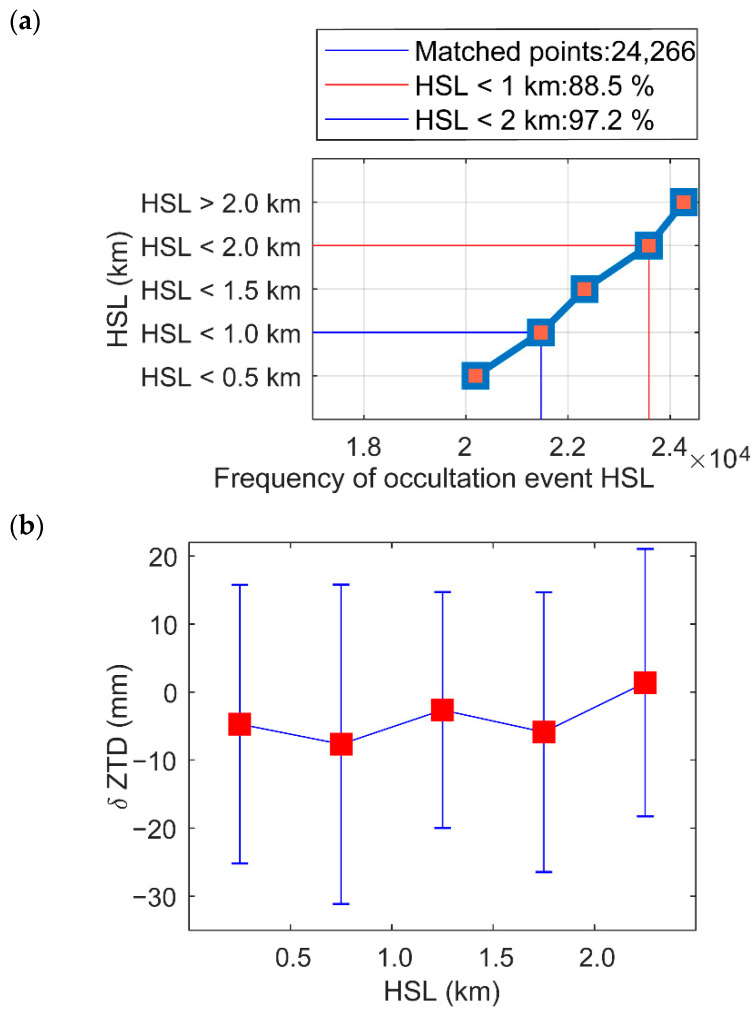
HSL variations in (**a**) frequency; and (**b**) residual ZTDs of matched atmPrf and gnsZTD in 2008.

We used ${\delta \mathrm{ZTD}}_{3}$ obtained by a filter with a horizontal distance of 300 km and a vertical distance of 1 km to compare and analyze the relationship between ${\delta \mathrm{ZTD}}_{3}$ and HSL (km). Figure 10a shows the range of GPS station heights and the amounts of matched occultation data. Figure 10a shows that the HSL data within 1 km reached 88.5% of observations, and the GPS station height within 2 km was close to 97.2%. Figure 10b shows the ${\delta \mathrm{ZTD}}_{3}$ statistics corresponding to different values of HSL. When δh is less than 1 km, the bias and standard deviation fluctuate as the station height increases, although gnsZTD is related to the station height, temperature, and water vapor. The absolute value of the bias is less than 10 mm, and the standard deviation is less than 25 mm. There is no significant trend in HSL.

**Figure 11:**
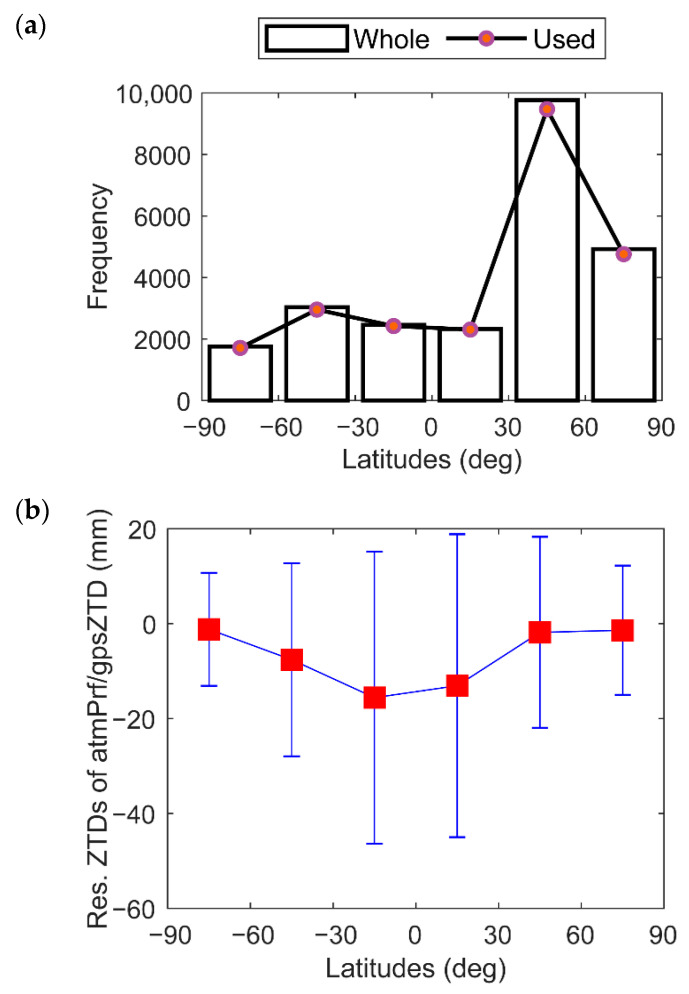
Latitudinal variations in (**a**) occultation event frequency; and (**b**) residual ZTDs of matched atmPrf and gnsZTD in 2008.

Figure 11 shows the total and used amounts, and statistics of ${\delta \mathrm{ZTD}}_{3}$ data in each latitude region for reducing the gross error by the 3-sigma principle to calculate the regional errors at different latitudes. Figure 11a shows that the data are mainly distributed in the mid-latitude region of the Northern Hemisphere (approximately 40%). Figure 11b shows the variation in ${\delta \mathrm{ZTD}}_{3}$ with latitude. In the northern and southern high-latitude areas (latitude greater than 60°), due to the low temperature and water vapor, the ${\delta \mathrm{ZTD}}_{3}$ biases are extremely small, and both are less than 5 mm; the standard deviations are less than 15 mm. As the latitude decreases, the temperature and water vapor increase, as well as ${\delta \mathrm{ZTD}}_{3}$. In particular, the bias near the equator is more than 10 mm, and the standard deviation is more than 30 mm. In addition, the bias and standard deviation of the ZTD near the mid-low latitudes in the Northern Hemisphere are slightly smaller than its bias and standard deviation in the Southern Hemisphere, which shows that the gnsZTD in the Northern Hemisphere performs better than that in the Southern Hemisphere.

## 4. Conclusions

We obtained ZTDs (occZTD, sonZTD, echZTD) by integrating refractivity from atmPrf, sonPrf, and echPrf from CDAAC/COSMIC, and gnsZTD derived from ground-based GNSS-ZTD. The statistics in 2008 showed that the absolute values of the bias between occZTD and echZTD, sonZTD, or gnsZTD are less than 5 mm, and the standard deviations are approximately 20 mm or less. The STDs in 2012 are slightly higher than that in 2008. For the temporal and spatial inconsistency between occZTD and sonZTD, the STD of ${\delta \mathrm{ZTD}}_{2}$ is greatly higher than that of ${\delta \mathrm{ZTD}}_{1}$. Since ${\delta \mathrm{ZTD}}_{3}$ includes errors from the correction method and matching constraints, its bias and STD have increased compared to ${\delta \mathrm{ZTD}}_{2}$. The spatial characteristics of the differences between ZTDs derived from COSMIC RO data and ZTDs from ECWMF and GPS-ZTD are discussed:

1. In the Northern and Southern Hemispheres, ${\delta \mathrm{ZTD}}_{1}$ increases with latitude; the ECMWF model can match the RO observation data better in the Northern Hemisphere than in the Southern Hemisphere; generally, the accuracy on land is higher than that in the marine region.
2. The accuracy of ${\delta \mathrm{ZTD}}_{3}$ increases as the horizontal or vertical matching threshold value increases.
3. The relationship between the accuracy of ${\delta \mathrm{ZTD}}_{3}$ and the altitudes of GPS stations is not obvious. However, similar to ${\delta \mathrm{ZTD}}_{1}$, ${\delta \mathrm{ZTD}}_{3}$ increases from high to low latitudes in the Northern and Southern Hemispheres; the gnsZTD can match the occultation observation data better in the Northern Hemisphere than in the Southern Hemisphere.

This work assesses ZTD derived from COSMIC occultation data with data from ECWMF, radiosondes, and GNSS. The accuracy of ${\delta \mathrm{ZTD}}_{3}$ at different horizontal distances from GPS stations can be used to evaluate the applicable region of occultation data and quantify the attenuation performance of occultation data for tropospheric delay errors in dynamic high-precision positioning, while the richness and uniformity of the occultation events to horizontal and vertical distances are related to the number and orbital design of low-orbit satellites (LEO) equipped with occultation equipment. COSMIC-2’s satellite can capture a lower level of atmospheric information with a higher gain of occultation antennas than that of COSMIC-1 and provide more atmospheric occultation observation data for its support to GPS/GLONASS constellations [31]. We look forward to better verification results from the atmosphere RO products from COSMIC-2 and other LEO constellations.

## Figures and Tables

**Table 1:** Statistics of differences between occZTD and echZTD, sonZTD and gnsZTD.

	Year	echZTD	sonZTD	gnsZTD
Bias/mm	2008	−2.3	0.8	−4.6
STD/mm		9.0	17.6	20.5
Bias/mm	2012	−1.2	0.3	−11.9
STD/mm		10	18.7	25.0

## Data Availability

The COSMIC atmosphere profiles used in the present work can be downloaded from the website https://cdaac-www.cosmic.ucar.edu/cdaac/products.html (accessed on 1 January 2021). The ZTD product and meteorological observations from IGS used in the present work can be downloaded from the website https://cddis.nasa.gov/archive/gnss/products/troposphere/rpt/ (accessed on 15 January 2021) and https://cddis.nasa.gov/archive/gnss/data (accessed on 15 January 2021), respectively.
